# Post-Irradiation Breast Angiosarcoma: All the Possible Treatments and Electrochemotherapy. Case Report and Literature Review

**DOI:** 10.3390/jcm13020567

**Published:** 2024-01-18

**Authors:** Simona Parisi, Claudio Gambardella, Francesco Iovino, Roberto Ruggiero, Francesco Saverio Lucido, Giusiana Nesta, Salvatore Tolone, Luigi Brusciano, Francesca Fisone, Federico Maria Mongardini, Giovanni Cozzolino, Carminia Maria Della Corte, Stefania Napolitano, Michele Orditura, Rosetta Esposito, Ludovico Docimo

**Affiliations:** 1 Department of Advanced Medical and Surgical Sciences, School of Medicine, University of Campania “Luigi Vanvitelli”, 80131 Naples, Italy; claudio.gambardella2@unicampania.it (C.G.); roberto.ruggiero@unicampania.it (R.R.); francescosaverio.lucido@unicampania.it (F.S.L.); salvatore.tolone@unicampania.it (S.T.); luigi.brusciano@unicampania.it (L.B.); ludovico.docimo@unicampania.it (L.D.); 2 Division of General, Oncological, Mini-Invasive and Obesity Surgery, University of Campania “Luigi Vanvitelli”, 80131 Naples, Italy; francesco.iovino@unicampania.it (F.I.); giusiana.nesta@gmail.it (G.N.); francesca.fisone@unicampania.it (F.F.); federicomaria.mongardini@unicampania.it (F.M.M.); giovanni.cozzolino@unicampania.it (G.C.); rosetta.esposito@unicampania.it (R.E.); 3 Department of Translational Medical Sciences, Division of General Surgery, University of Campania “Luigi Vanvitelli”, 80131 Naples, Italy; 4 Medical Oncology, Department of Precision Medicine, University of Campania “Luigi Vanvitelli”, Via S. Pansini 5, 80131 Naples, Italy; carminiamaria.dellacorte@unicampania.it (C.M.D.C.); stefania.napolitano@unicampania.it (S.N.); michele.orditura@unicampania.it (M.O.)

**Keywords:** breast angiosarcoma, post-irradiation angiosarcoma, electrochemotherapy

## Abstract

Breast angiosarcoma is a rare malignancy, accounting for less than 1% of all soft tissue cancers. It comprises primitive and secondary subtypes, such as radiogenic breast angiosarcoma (RAS). Despite multimodal treatment, angiosarcomas represent an incurable disease for many patients and a significant cause of deterioration in their quality of life. Surgery is a cornerstone in management, but high recurrence rates are reported. Electrochemotherapy (ECT) is a practicable locoregional treatment for patients with advanced angiosarcoma as part of a multimodal therapeutic strategy. The palliative benefits of ECT include optimal patient compliance, good local hemostasis control, and positive local responses. Since only 22 cases are described in the literature, we reported a rare case of RAS treated with ECT after a multidisciplinary approach, including Next Generation Sequencing (NGS). A literature review on the feasibility of ECT in RAS management was also performed.

## 1. Introduction

Breast angiosarcoma is a rare malignancy, accounting for less than 1% of all soft tissue sarcomas [1]. It originates from the aberrant proliferation of endothelial cells in small-blood or lymphatic vessels. The most well-known microscopic features include nuclear atypia, frequent mitosis, hyperchromatic nuclei, and large nucleoli [2].

Primitive angiosarcoma comprises only 0.04% of all breast malignancies [3] and manifests as an intraparenchymal enlarging mass. It predominantly affects younger women, and diagnosis is often delayed due to its rarity and gland density [4]. In contrast, secondary angiosarcoma typically affects older women and involves the cutaneous layers. It is associated with lymphedema conditions such as Stewart and Treves Syndrome, which is a complication of post-demolitive breast surgery. However, the widespread adoption of breast-conservative surgery has not reduced the risk of breast angiosarcoma, as post-irradiation subtypes are correlated with adjuvant radiotherapy (RT) [5].

Primitive and radiogenic angiosarcomas (RAS) are morphologically indistinguishable, but pathogenetic differences are evident. For instance, cellular-MYC (c-MYC) amplification is strongly associated with post-irradiation subtypes and not with primitive subtypes, suggesting a specific oncogenic pathway and a potential targeted therapy [6].

The extreme rarity of this condition limits the possibility of clinical trials to assess the most adequate treatment strategy; therefore, decisions are based on retrospective case reviews [7].

Moreover, surgery is the cornerstone for the treatment of breast angiosarcomas, and the most important prognostic factor is the status of margins [8]. The roles of radiotherapy (RT) and adjuvant chemotherapy (CT) are unclear, and guidelines are not available. In the 2000s, electrochemotherapy (ECT) was proposed as a potential treatment for superficial malignant lesions. The first evaluation of its therapeutic role in angiosarcoma management was conducted in 2001 in veterinary medicine, achieving better results than cisplatin treatment alone [9]. ECT is gaining recognition as an effective local therapy based on the systemic or local injection of bleomycin or cisplatin combined with electroporation.

This technique involves the administration of electric pulses that permeabilize cells in the exposed tissue. The resulting cellular pores increase the uptake of hydrophilic chemotherapeutics that face challenges during transport through the cell membrane [10]. ECT offers many advantages, such as an enhanced drug concentration in neoplastic cells and a reduction in general cytotoxic effects [11]. Furthermore, the use of electrochemotherapy is widespread, and numerous studies have evaluated its effectiveness in angiosarcoma management [12].

Despite multimodal treatment, angiosarcomas remain incurable for many patients and contribute substantially to the deterioration of their quality of life. Genomic molecular characterizations, such as Next Generation Sequencing (NGS), often conducted to investigate tumor aggressiveness and potential experimental therapeutic choices, can also be performed to assess the therapeutic responsiveness of traditional treatments and the potential use of ECT. 

We present a case of a 57-year-old woman affected by post-irradiation breast angiosarcoma treated with ECT and provide a literature review on the impact of ECT on angiosarcoma therapies.

## 2. Case Presentation

Before obtaining informed written consent, we present the case of a 57-year-old woman with a history of atrial fibrillation and a diagnosis of invasive ductal carcinoma of the breast in 2014. She underwent breast-conserving surgery, radiotherapy (RT), and hormonal therapy (HT) with Femara. The initial cancer was classified as early-stage (pT1c N0 M0), expressing estrogen and progesterone receptors (ER = 90%, PgR = 30%), a moderate proliferation index (Ki-67 = 30%), and a non-amplified c-ERB-2 gene. The patient had a quadrantectomy, axillary lymph node sampling, and received standard RT protocols (60 Gy in 30 fractions).

**Figure 1:**
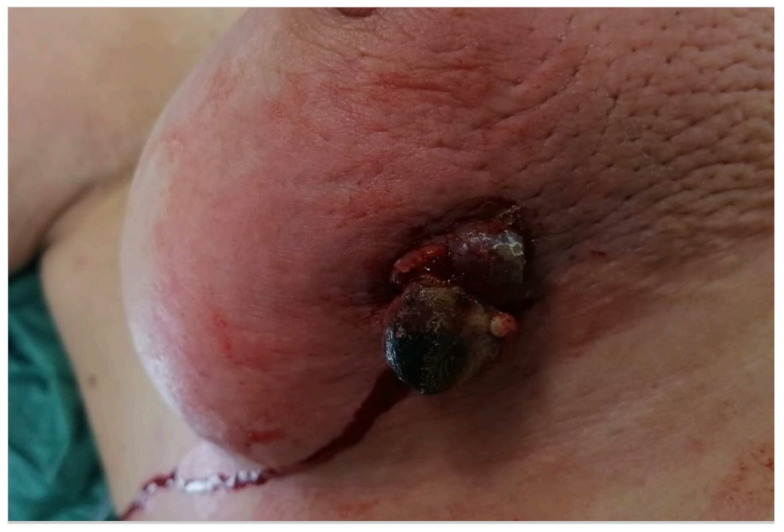
Post-irradiation breast angiosarcoma before the surgery.

Five years later, the patient noticed an exophytic lump in the skin corresponding to the lower lateral quadrant of the right breast (Figure 1). Initially dismissed as a pimple, she became concerned when it increased in size over six months. A dermatological examination revealed a 20 mm lesion without pain, but with erythema and an orange peel skin appearance. During the medical visit, the lesion began to bleed, raising suspicion of a breast SBA (Figure 1). The patient was referred to our General Surgery Unit at Campania University “Vanvitelli” in Naples, Italy.

We recommended bilateral mammography and breast ultrasound (US), but the patient could not tolerate compression on the right breast. Consequently, only breast US was performed, revealing a 22 mm exophytic lesion with a hypoechoic pattern, irregular margins, and rich peri- and intra-tumoral vascularization. A surgical biopsy confirmed the suspicion of breast angiosarcoma, intermediate grade according to the French Federation of Cancer Centers (FNCLCC) [13].

The “Vanvitelli” Oncological Multidisciplinary Group (OMG) recommended radical right mastectomy and instrumental follow-up. The surgery was performed in April 2020. Genomic molecular characterization using Next Generation Sequencing (NGS) was performed on the tumor sample to explore potential experimental therapeutic options. No targetable alterations were detected, but the tumor exhibited mutations in the NF1 gene and MYC amplification, indicating biological aggressiveness. The tumor was also microsatellite-stable (MSS) with low tumor mutational burden (TMB), indicating a negative profile for immune-responsiveness.

**Figure 2:**
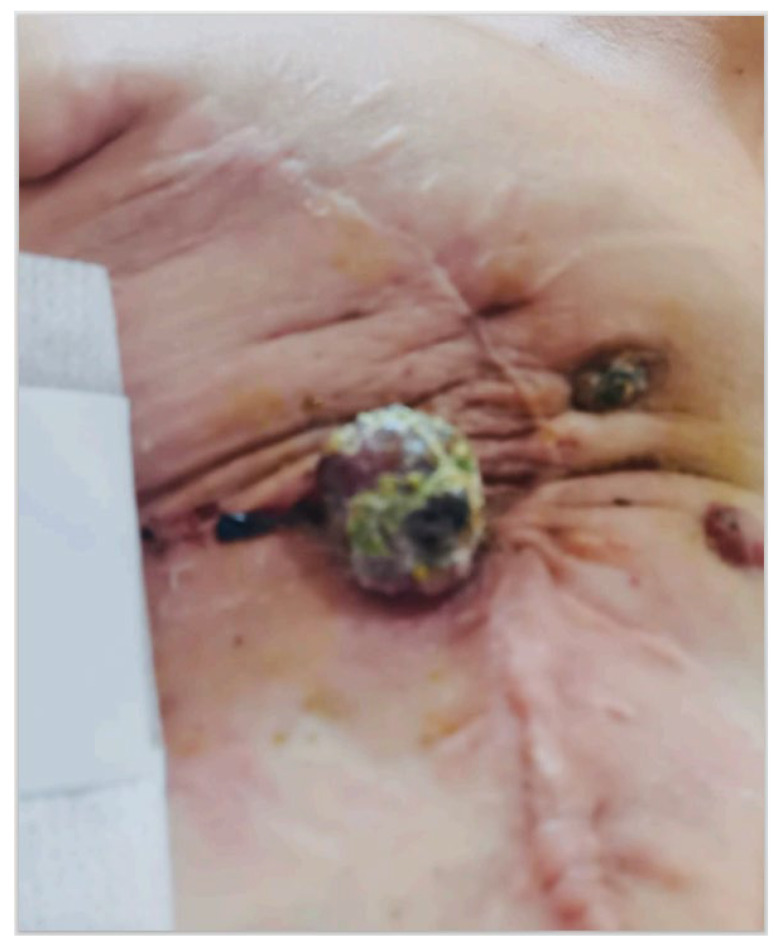
Relapsed post-irradiation breast angiosarcoma after the mastectomy.

Eighteen months later, new purple skin lesions appeared in the anterior right thoracic region, and an 18-fludeoxyglucose–Positron Emission Tomography (18-FDG-PET) revealed pectoralis muscle involvement (Figure 2). The patient underwent 14 cycles of Paclitaxel (Doxorubicin was contraindicated due to atrial fibrillation comorbidity) from February 2022 to January 2023, and re-irradiation (40.5 Gray (Gy) for 15 cycles). Despite the treatments, new lesions spread to thoracic and abdominal skin regions, and a neoplastic pulmonary effusion was diagnosed. The OMG recommended thoracentesis, ECT, and Gemcitabine treatment.

**Figure 3:**
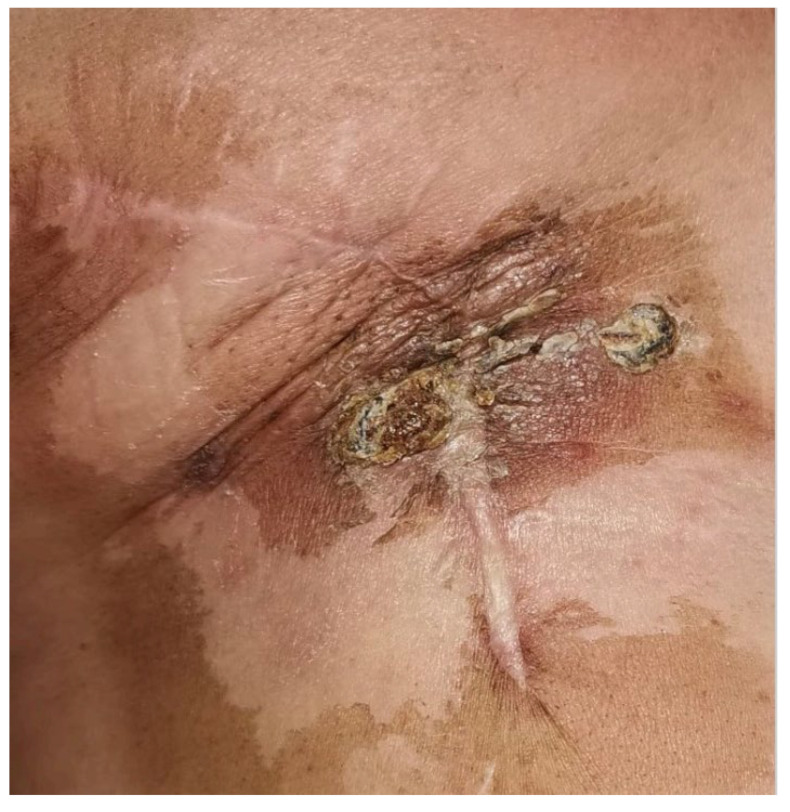
Post-irradiation breast angiosarcoma after the Electrochemotherapy.

Unfortunately, the patient could not tolerate Gemcitabine due to the development of a severe skin rash. She underwent three ECT sessions, receiving Bleomycin at the standard regimen of 15,000 IU through intravenous injection during deep sedation. After thorough skin disinfection, electrodes were applied, and the procedures lasted about 40 min with no complications. Bleeding lesions underwent careful hemostatic control, and ECT cycles were performed monthly in day surgery hospitalization (Figure 3).

The patient developed a severe neoplastic pulmonary effusion, leading to the cessation of ECT due to lung contraindication. Local lesions improved with frequent bleeding and infections. Unfortunately, the patient succumbed to respiratory failure 24 months after the diagnosis of RAS.

## 3. Literature Review

In a systematic review literature search of the database PubMed (search date 10 February 2023) using the search terms “angiosarcoma and electrochemotherapy”, we identified only 14 citations [9,12,14,15,16,17,18,19,20,21,22,23,24,25]. Seven studies were excluded because two of them were commentary [15,16], one referred to the animal population [9], another was about melanoma or non-melanoma diseases [20], two were about the Stewart–Treves Syndrome [18,19,20,21], and one was not about RAS [22]. Thus, only seven studies reported cases of RAS also treated with ECT. Twenty-two cases were reported in the literature. 

The clinical studies describing patients with RAS of the breast also treated with ECT are reported in Table 1. The most affected age is between 61 and 77 years, with a latency period from the irradiation of 48–108 months. Only six studies reported the histology of the previous breast cancer, and luminal subtypes are prevalent (6/6 cases reported). All the patients received a conservative surgical approach (quadrantectomy), except a patient who underwent mastectomy [14]. Only two women received adjuvant CT. All the patients (6/6) were treated with hormonal therapy and adjuvant RT. Only in one case, 25 Gy were administered, while the other patients received 60 Gy in total in 30 fractions. 

The most frequent clinical sign of RAS was a red-purple nodular near the surgical scar or on the same breast. In 22/23 cases, the patients underwent surgery, while only 8/23 received a new CT, with doxorubicin as the first choice in almost 3 cases. Docetaxel, paclitaxel, and gemcitabine were considered when doxorubicin was contraindicated. Only 12/22 underwent a re-irradiation. 

Twenty-two patients received ECT for 1–8 cycles (mean 1.35 cycles per patient). Bleomycin 15.000 UI was the favorite treatment, and common adverse reactions were pain, edema, ulceration, and necrosis. Complete and partial responses (CRs and PRs) were reported for 7/22 and 8/22 patients, respectively. Overall survival (OS) was 3–29.9 months (mean value: 4.1 months), while free progression survival (FPS) was 1–24 months (mean value: 3.6 months).

## 4. Discussion

Breast cancer is the most prevalent cancer in women, and its incidence continues to rise annually. Current treatment modalities include surgical resection, chemotherapy (CT), radiotherapy (RT), and hormone therapy. Approximately one-third of lesions are estimated to be non-palpable cancers, leading to an increasing preference for conservative surgery followed by RT [26,27,28,29].

Electrochemotherapy (ECT), which exposes tumor cells to electric pulses through a process known as electroporation, in combination with cytostatic drugs, appears to be a safer and more effective treatment for breast cancer in both in vitro and in vivo settings. Indeed, this approach has found applications in the treatment of breast cancer and its metastases. Furthermore, palliative effects have been established, with noted reductions in pain for patients. Its utilization has also been extended to lesions related to radiogenic angiosarcomas (RASs).

It behaves aggressively and carries a poor prognosis, with a 5-year overall survival rate of 27–48%. Due to its scarcity, most of the information regarding the diagnosis and management of this condition is from case reports and retrospective series analyses with relatively small patient numbers. RAS is even less common [30].

ECT is now also recognized as a loco-regional therapy for disseminated cutaneous and subcutaneous tumor lesions, improving the patient’s quality of life. The safety and efficacy of ECT for primary basal cell carcinoma and primary squamous cell carcinoma, which are the most common types of skin cancer, are well known. 

Surgery represents the main treatment for RAS as a diagnostic and therapeutic procedure. However, it is particularly challenging for advanced and multifocal types and is often associated with recurrences. A delayed diagnosis is an important concern. Initial skin changes in radiogenic angiosarcoma are subtle; therefore, these alterations may be confused with other benign skin conditions such as telangiectasia. In these situations, CT re-irradiations are also performed, even if the drug regimen is empirical and no standard regimen is approved for RAS. However, doxorubicin and taxanes are considered the most common option in patients with metastatic angiosarcomas, and the overall response rate has been estimated at 30%. Unfortunately, it has been verified that complete responses are about 6%, partial responses 29.5%, and stable responses 13% [31,32]. In a recent review, Depla et al. evaluate the effect of treatment modalities surgery, RT, and CT on RAS patients. They focused on a high recurrence rate after surgery (65%, of which 91% were local). Better results were obtained when surgery was combined with RT (51%, of which 68% were local), while no improvement was identified in the group treated with surgery and CT [33]. Despite the multimodal treatment, angiosarcomas represent an incurable disease for many patients and represent a cause of substantial deterioration of their quality of life. Thus, the introduction of the most recent technologies is strongly encouraged.

ECT has demonstrated clinical utility in the management of superficial cancer, and it has been associated with better tumor control and patients’ outcomes [34,35,36,37,38]. One of the larger studies about ECT in angiosarcoma treatment was performed by Campana et al. Twenty patients received 24 ECT courses at the standard regimen of Bleomycin 15.000 UI, and 51 lesions were treated. The mean duration of the procedures was 28 min, and 15% of the patients were candidates for other systemic therapies. Considering the per-tumor local response, they obtained the following results: CR = 61%, PR = 22%, and disease stabilization (SD) = 18%. The per-patient response was CR = 40%, PR = 40% and SD = 15%. Unfortunately, 35% of the patients experienced local recurrence after a median period of 3.4 months. The main adverse reactions reported were pain for 30% of the patients and skin toxicity with ulceration (25%) [12] (Table 2). 

Our clinical case was characterized by a partial response to ECT courses, but skin toxicity was elevated. Unfortunately, the patient developed lung metastasis with respiratory failure, preventing further treatments and limiting ECT effectiveness. During lung center hospitalization, the patient reported an evident progression of local lesions, with difficult management of bleeding and infected areas. 

Contrary to other skin cancers (such as Kaposi’s sarcoma) and breast cancer, clinical experience with ECT in angiosarcoma is still limited and is accepted as a palliative strategy. However, some case reports are suggesting the feasibility of ECT. For example, Benevento et al. reported the case of a 76-year-old woman treated with a multimodal approach (breast conservative surgery, RT, CT, and ECT), focusing on the complete local response in all the lesions and improving life quality [17].

Despite the multimodal therapeutic approach, angiosarcomas represent an incurable disease for many patients. Genomic molecular characterizations could be routinely performed to investigate the aggressiveness of the tumors and to predict the therapeutic responsiveness of traditional treatments. A more aggressive phenotype could be a candidate for close follow-up and for earlier ECT cycles. 

Also, genomic molecular characterizations such as NGS (next-generation sequencing), which are often performed to investigate the aggressiveness of tumors and potential experimental therapeutic choices, may be performed to evaluate the therapeutic responsiveness of traditional treatments and the possible use of ECT. 

For most sarcomas, the driver mutations remain unknown. Cote et al. described their experience with NGS, retrospectively analyzing the results of 133 cases. An average of two gene alterations were identified per tumor sample, and eighty-eight percent of samples had at least one detected mutation. There were 75 mutations in genes that are targetable with existing drugs. Further studies could determine if these mutations are clinically meaningful drug targets in sarcoma [35]. MYC amplifications have been frequently detected in RAS. However, NGS was performed to investigate the genomic landscape of RAS. Dermawan et al. performed a detailed comparative genomic investigation of RAS versus other RT-associated histotypes. RAS had more frequent MYC, FLT4, CRKL, HRAS, and KMT2D alterations than sporadic angiosarcomas [36]. Cote et al. conducted a study to verify whether c-MYC amplification can reliably discriminate RAS and primary breast angiosarcoma. They demonstrated that the amplification (5- to 20-fold) of the c-MYC oncogene was found in all breast RASs and in none of the primary angiosarcomas except one. These data showed the pathways preferentially involved in the pathogenesis of RAS of the breast and may provide the basis for an additional targeted therapy [35].

In our clinical case, NGS was performed to verify the tumor’s mutations and to evaluate biological aggressiveness and the profile for immune-responsiveness. No targetable alterations were detected, but the tumor exhibited mutations in the NF1 gene and MYC amplification, indicating biological aggressiveness. The tumor was also microsatellite-stable (MSS) with low tumor mutational burden (TMB), indicating a negative profile for immune-responsiveness.

MYC amplification has been reported as a prominent feature of RAS. Survival analysis of RAS patients demonstrates that those with MYC amplification had a significantly worse overall survival compared to cases without MYC amplification (*p* = 0.035). MYC amplification was associated with adverse prognosis, suggesting a prognostic role in RAS of the breast [36].

Strategies to target the cell cycle by Cyclin-dependent Kinase (CDK) inhibition have shown promising results in MYC-amplified tumors. In neuroblastoma cell lines, CDK/CDK1 inhibitors show an ability to downregulate MYC. Recently, fadraciclib, a potent inhibitor of CDK9, showed an ability to repress MYC and is currently in early-phase clinical trials for solid tumors and hematologic malignancies [37,38].

The reported experiences indicate that ECT is a practicable locoregional treatment in patients with advanced angiosarcoma, in a multimodal therapeutic strategy. In the case reported by Cencelj-Arnez, ECT was performed in a breast reconstructed by autologous free-flap. Only four similar cases were reported in the literature, describing RAS after mastectomy, autologous breast reconstruction and adjuvant RT. The technique was feasible, safe and effective in treatment of the disease. Most of the treated lesions reached a complete response in several consecutive ECT sessions, but multiple recurrences occurred in non-treated areas [14]. ECT may represent one of the feasible options in the management of RAS. Patients undergoing skin-sparing mastectomy and immediate breast reconstruction who receive RT should be monitored by regular long-term follow-up, and the biopsy of any suspicious lesions is mandatory. 

The use of ECT could also be realized in the case of prosthetic reconstruction. Campana et al. reported the case of a 55-year-old woman with previous skin-sparing mastectomy and prosthetic reconstruction for multifocal ductal carcinoma who developed homolateral axillary recurrence. After the surgery, the pathology report revealed a positive cutaneous margin. Since further breast skin excision or RT would have compromised the prosthetic implant and the patient rejected further surgical or RT therapies, the multidisciplinary recommendation included ECT. The procedure lasted 20 min under mild general sedation and included a bolus of intravenous bleomycin followed by local application of electric pulses using a needle electrode. There were no postprocedural complications. At the 5 year follow-up, the patient is disease-free with the implant in situ. This report illustrates the role of ECT in sterilizing resection margins and preserving a breast implant [34].

CT is becoming an increasingly challenging antitumor therapy to administer due to the numerous mechanisms of drug resistance. To address this issue, alternative techniques such as ECT can be employed. The latter involves the simultaneous administration of electrical pulses (electroporation) and drug treatment to enhance the drug’s effectiveness against the tumor. Promising results have been observed in in vitro studies, veterinary applications, and clinical oncology research, particularly in various cancers such as metastatic melanoma.

All the reported cases described a local improvement, and three studies reported complete responses. ECT is also useful as a palliative option because it is associated with optimal patient compliance, good local hemostasis control, and infection prevention. Other future studies are necessary to establish the optimal timing, the extension of treatment, and the most appropriate combination of surgery and systemic treatments. Thus, genomic molecular characterizations may play a role in RAS management. 

Moreover, ECT has emerged as a promising palliative treatment, raising interest in exploring its combination with RT to enhance tumor response. However, the potential benefits and challenges of combining these treatments remain unclear, even if their combination consistently improved tumor response when compared to individual therapies [39].

## 5. Conclusions

Although surgery represents the cornerstone in the management of RAS, high recurrence rates are reported. NGS can be performed to investigate the aggressiveness of the angiosarcoma and to evaluate all the potential therapeutic choices. ECT is a practicable locoregional treatment in patients with advanced angiosarcoma, realizing a local improvement of lesions or a palliative management of complications. Other possible applications include skin lesions in patients undergoing reconstructive breast surgery.

The reported case emphasizes how angiosarcoma represents a condition that is capable of causing a significant deterioration in the quality of life and is associated with high mortality due to recurrences. Surgery remains the fundamental treatment, but the use of electrochemotherapy could be considered in connection with it in order to target recurrences. Timing is crucial and should be carefully evaluated. Furthermore, the implementation of Next-Generation Sequencing (NGS) could be useful to explore all possible therapeutic options and predict cases associated with higher aggressiveness, where recurrence is more likely and where ECT could be planned early.

Further studies are necessary to establish the timing, the extension of treatment, and the possible role of genomic characterizations in RAS management and patients’ selection.

## Figures and Tables

**Table 1:** Clinical studies describing patients with radiogenic angiosarcoma (RAS) of the breast treated using electrochemotherapy (ECT).

Study	Type of Study	Patients	Age	History	First Treatment	Latency Period Months	Clinical Features	Skin Region	Re-Treatment	Histology	C myc Amplification
Cencelj-Arnez 2020 [14]	CR	1	63	Syncronus bilateral Luminal BC (right breast)	Mastectomy + 6 cycles x 5-fluorouracil, epirubicin, Cyclophosphamide + letrozole + RT 25Gy	60	Ulcerated red lesion	Lower-medial quadrant In right breast	Excision + ECT + doxorubicin	High grade RAS	Yes
Campana 2019 [12]	CS	20 (10 breast RAS)	/	/	/	/	/	/	/	/	/
Benevento 2015 [17]	CR	1	76	Invasive ductal carcinoma Luminal BC (left breast) pT1 pN0 M0 G2	BCS + 50 Gy in 25 fractions of 200 cGy/daily with boost of 10 Gy in 5 fractions of 200 cGy/daily + Tamoxifene	48	painful, violet, multi-nodular mass	Left > right breast	Excision + Mastectomy (after 4 years) + doxorubicin	grade-II RAS	/
Guida 2016 [19]	RS	19 (6 breast RAS)	69	/	/	96	/	Scalp (5) Breast (8) Skin (3) Soft tissue (3)	ECT (19/19)+ Surgery (17/19) + RT (5/19) + CT (3/19)	RAS	/
Mocerino 2016 [23]	CR	1	77	invasive ductal carcinoma pT1N0M0 ER + 15%; PgR + 30%; HER2 IHC 1 + (left breast)	BCL + 60 Gy in 30 fractions + tamoxifen	84	ecchymotic lesion (1.3 cm)	near the scar	Excision + left mastectomy (after 1 year) + right mastectomy (after 2 years) + ECT + 69 Gy + Doxorubicin	low-grade RAS	/
Laurino 2022 [24]	CR	1	61	infiltrating ductal carcinoma, pT1cN0, grade G2, ER 98%, PGR 20%, HER2 +, left breast	BCL + 50 Gy in 25 fractions + 10 Gy in 5 fractions by photons + Adjuvant CT + letrozole	72	/	Left breast	Neoadjuvant CT + mastectomy (after 1 year) + ECT + Re-excision	high-grade RAS (G3), positive for Factor VIII and CD31, with extensive areas of necrosis and ulceration.	/
Laurino 2022 [24]	CR	1	63	infiltrating ductal breast cancer pT1cN1(1/18), G2, ER: 90%, PGR: 60%, Ki67 index at 15%, and HER2 negative Left breast	BCS+ 5-fluorouracil, epidoxorubicin, and cyclophosphamide+ 50 Gy in 25 fractions + 10 Gy in 5 fractions by photons+ letrozole	108	ulcerated and bleeding left breast lump, 7 cm in diameter, adherent to the chest wall	Left breast	Radiofrequency termoablation + gemcitabine and docetaxel + ECT +	RAS	/
Current case report	CR	1	59	breast invasive ductal Luminal B carcinoma pT1c N0 M0 (right breast)	BCL + 60 Gy in 30 fractions+ femara	60	exophytic lump	near the scar	Excision + right mastectomy + Paclitaxel (doxorubicin contraindicated) + 40.5 Gy in 15 fractions + ECT	Grade II RAS	/

CR = case report, CS = cohort study, RS = retrospective study.

**Table 2:** Electrochemotherapy modalities and results.

Study	ECT						
	Cycles	Drug	Dose	Adverse Reactions	Overall Survival	Free-Progression Survival (Months)	Results
Cencelj-Arnez 2020 [14]	3	Bleomicin	30,000	edema	19	1	CR = 100%
Campana 2019 [12]	24 (10 Breast RAS; 1 ECT per patient)	Bleomicin	250–1000 IU/cm^3^ or 15,000/m^2^	Skin ulceration (25%) pain (30%)	12.5	1.8	CR 40% (8/20) PR, 40% (8/20)
Benevento 2015 [17]	8	Bleomicin	15,000 IU/m^2^	/	18	18	CR = 100%
Guida 2016 [19]	/	Bleomicin	15,000 IU/m^2^	Pain	29.9	/	CR = 42% PR = 21%
Mocerino 2016 [23]	2	Bleomicin	15,000 IU/m^2^	/	21	21	CR = 100%
Laurino 2022 [24]	/	/	/	/	3	3	Local condition improvement
Laurino 2022 [24]	2	/	/	/	24	24	Local condition improvement
Parisi 2023	3	Bleomicin	15,000 IU/m^2^	Pain Edema necrosis	24	17	Local condition improvement

## Data Availability

The datasets used and/or analysed during the current study are available from the corresponding author on reasonable request.

## Abbreviations

RAS: radiogenic angiosarcoma; ECT: electrochemotherapy; NGS: next-generation sequencing; RT: radiotherapy; c-MYC: cellular-MYC; CT: chemotherapy; ERs: estrogen receptors; PRs: progesterone receptors; US: ultrasound; FNCLCC French Federation of Cancer Centers; OMG: oncologic multidisciplinary group; 18-FDG-PET: 18-fludeoxyglucose–Positron Emission Tomography; Gy: Gray; FPS: free progression survival; CRs: complete responses; PRs: partial responses; SD: disease stabilization.
